# Learning an unknown transformation via a genetic approach

**DOI:** 10.1038/s41598-017-14680-7

**Published:** 2017-10-30

**Authors:** Nicolò Spagnolo, Enrico Maiorino, Chiara Vitelli, Marco Bentivegna, Andrea Crespi, Roberta Ramponi, Paolo Mataloni, Roberto Osellame, Fabio Sciarrino

**Affiliations:** 1 Dipartimento di Fisica, Sapienza Università di Roma, Piazzale Aldo Moro 5, I-00185 Roma, Italy; 2 Istituto di Fotonica e Nanotecnologie, Consiglio Nazionale delle Ricerche (IFN-CNR), Piazza Leonardo da Vinci, 32, I-20133 Milano, Italy; 3 Dipartimento di Fisica, Politecnico di Milano, Piazza Leonardo da Vinci, 32, I-20133 Milano, Italy

## Abstract

Recent developments in integrated photonics technology are opening the way to the fabrication of complex linear optical interferometers. The application of this platform is ubiquitous in quantum information science, from quantum simulation to quantum metrology, including the quest for quantum supremacy via the boson sampling problem. Within these contexts, the capability to learn efficiently the unitary operation of the implemented interferometers becomes a crucial requirement. In this letter we develop a reconstruction algorithm based on a genetic approach, which can be adopted as a tool to characterize an unknown linear optical network. We report an experimental test of the described method by performing the reconstruction of a 7-mode interferometer implemented via the femtosecond laser writing technique. Further applications of genetic approaches can be found in other contexts, such as quantum metrology or learning unknown general Hamiltonian evolutions.

## Introduction

Linear optical networks have recently received increasing attention in the quantum regime thanks to the enhanced capability of building complex interferometers made possible by integrated photonics. This experimental achievement opened new perspectives in the adoption of linear optical networks for different quantum tasks, including quantum walks and quantum simulation^1–10^, quantum phase estimation^11–13^, as well as the experimental implementation of the Boson Sampling problem^14–21^. Within these contexts, it becomes crucial to learn the action of a linear process. On one side, the capability of efficiently reconstructing an unknown transformation provides an analysis tool for integrated devices. Indeed, it allows to verify the quality of the fabrication by checking the adherence of an implemented transformation with the desired one. Conversely, on the fundamental side precise knowledge of the unitary process is required to perform accurate tests on the experimental data. For instance, this holds in the case of Boson Sampling validation, where the adoption of statistical tests may require knowledge of the implemented unitary transformation^19,20^. Furthermore, the task of learning an unknown transformation can be in principle embedded into a larger class of problems, whose objective is to learn physical evolutions from training sets of data^22–24^.

While initial efforts have been dedicated to the characterization of generic quantum processes^25–32^, different methods have been specifically adopted and tested to reconstruct an unknown linear transformation ${\mathscr{U}}$ ^33–37^. Most of these approaches rely on single-photon and two-photon measurements. Intuitively, single-photon states can be used to obtain information on the square moduli of the unitary matrix, while two-photon interference provides knowledge of the complex phases of the elements of ${\mathscr{U}}$. Different data analysis approaches have been proposed and adopted to convert the raw measured data in an estimated unitary ${{\mathscr{U}}}_{r}$, exploiting conventional numerical minimization techniques^33^ or by analytically inverting the relations between experimental data and the elements of ${\mathscr{U}}$ ^34^. Other methods exploit classical light as input in the interferometer^35^. In this case, knowledge on the moduli is obtained by sending classical light on a single input, while knowledge on the phases is obtained by sending light on pairs of input modes and by measuring the interference fringes as a function of the relative phase.

In this article we discuss and test experimentally an approach for the reconstruction of linear optical interferometers based on the class of genetic algorithms^38–40^. The latter is a general method that exploits the principles of natural selection in the evolution of a biological system, and has found application to find the solution to optimization and search problems in several fields, including first applications in quantum information tasks^41,42^. In these papers, genetic approaches have been adopted to determine the unitary transformation solving a given computational problem^41^, and to optimize the digital implementation of a given Hamiltonian from a set of imperfect gates within a quantum simulation framework^42^. Furthermore, genetic approach have also found successful application for quantum control tasks^43–45^. We first discuss the general principles of operations of the broad class of genetic algorithms. Then, we show how to adapt these principles of operations to the specific case of linear optical networks tomography. Finally, we test experimentally the genetic algorithm by performing the reconstruction of a *m* = 7 modes integrated interferometer built by the femtosecond laser-writing technique^46,47^.

## Results

### Genetic reconstruction algorithm for unitary transformations

Genetic algorithms are a broad class of algorithms inspired by the natural evolution of biological systems, which evolve following the principle of natural selection^38–40^. This principle can be briefly described as follows: within an ecosystem, individuals struggling for survival coexist within the same population. Genetically fittest individuals, e.g. those with highest adaption to environmental variables, are more likely to survive and reproduce. The fitness of an individual is determined by its genetic signature, the DNA, which is composed by a set of genes representing its fundamental units. The set of genes belonging to all the individuals of a given population is called genetic pool. Two individuals generate the offspring that inherits a combination of the genes belonging to both the parents by means of reproduction. Thus, a single gene is or is not inherited but cannot be partially inherited. If the combination of inherited genes determines a better fitness than the parents’ one, the son will have higher survival probability. Since weaker individuals are more unlikely to survive, fittest genes are more likely to spread over the population and, consequently, a gradual improvement of the average fitness of the population is expected. The described evolution, however, would be destined to reach a local maximum since the evolved genetic pool would be composed of just a subset of the initial one. Indeed, the mechanism of reproduction does not allow the creation of new genes. This would imply that the maximum possible fitness reached within the population would strongly depend on the initial state. Hence, it is crucial to consider in this model also the mechanism of mutation^38–40^, a rare event that manifests when an inherited gene changes in a random fashion. This mutated gene would likely not be present in any of the parents’ DNA and could possibly provide new advantageous features, causing the increase of the individual’s probability of survival. This will allow the mutated gene to spread over the population by reproduction, increasing the maximum fitness achievable within the given genetic pool and thus rendering the evolution no longer limited by the initial conditions.

**Figure 1:**
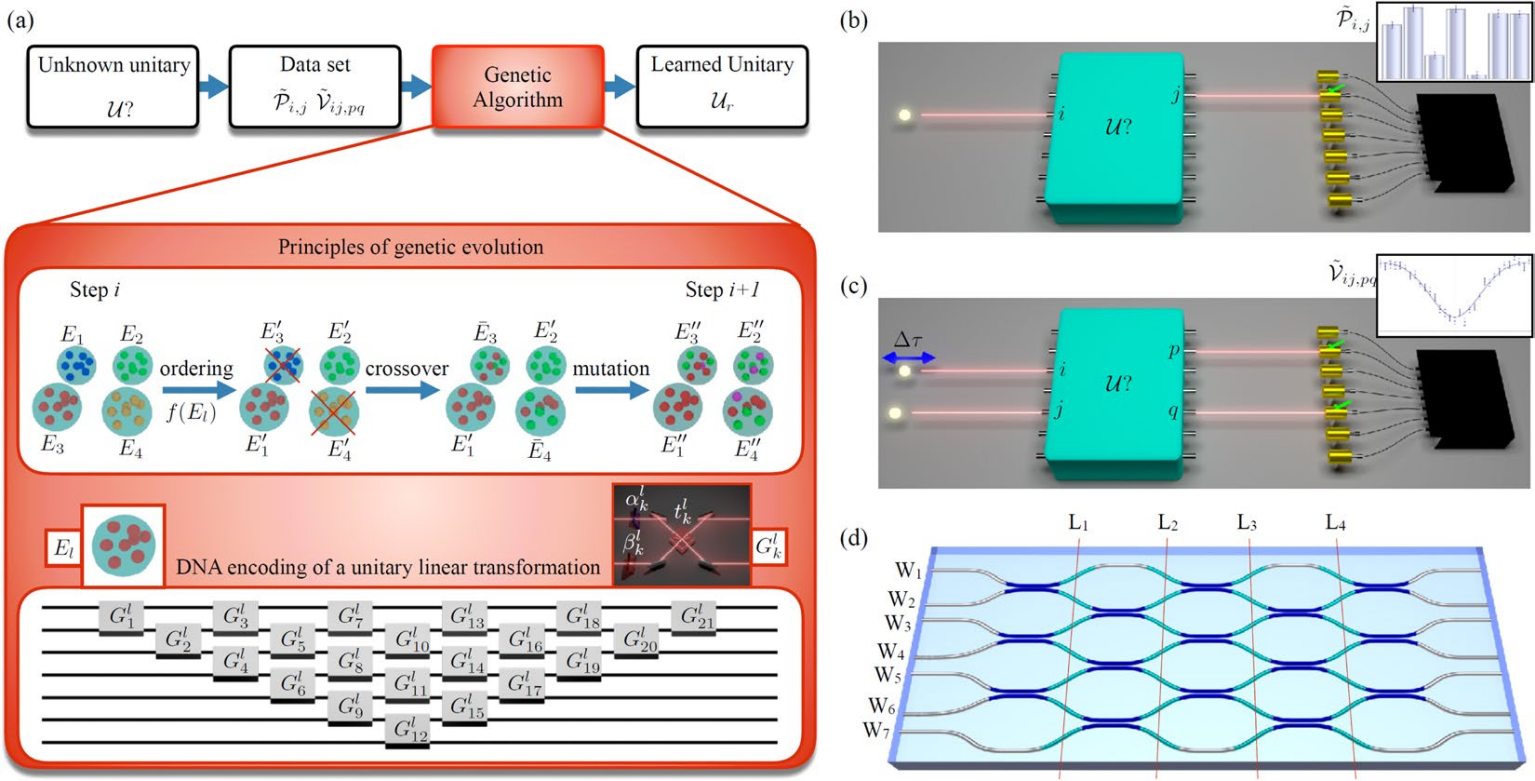
(**a**) Learning an unknown linear unitary transformation via a genetic approach. The training set, measured from the unknown transformation, is processed by an algorithm based on the principles of biological systems. The unitary transformation is decomposed in elementary units, i.e. the genes ${G}_{k}^{l}$ composing its DNA: beam-splitters (BSs) with transmittivity ${t}_{k}^{l}$ and phase-shifts (PSs) ${\alpha }_{k}^{l}$, ${\beta }_{k}^{l}$. Crossover and mutation mechanisms rule the evolution for each step of the algorithm. (**b**) Schematic view of single-photon measurements corresponding to data set ${\tilde{{\mathscr{P}}}}_{i,j}$. (**c**) Schematic view of two-photon measurements corresponding to data set ${\tilde{{\mathscr{V}}}}_{ij,pq}$. (**d**) Internal structure of the implemented *m* = 7 integrated linear interferometer. Blue regions indicate directional couplers, that is, integrated versions of beam-splitters, while cyan regions indicate phase shifts (4 layers L_1_–L_4_), introduced by modifying the optical path of the waveguides (W_1_–W_7_).

The principles of genetic algorithms can be applied to learning an unknown linear transformation ${\mathscr{U}}$ (Fig. 1a). The goal is to find the unitary matrix ${{\mathscr{U}}}_{r}$ whose action best describes a set of experimental data. The training set is composed of single-photon probabilities ${{\mathscr{P}}}_{i,j}$, describing the transition from input *i* to output *j* (Fig. 1b), and Hong-Ou-Mandel^48^ visibilities, describing two-photon interference from inputs (*i*, *j*) to outputs (*p*, *q*) (Fig. 1c). Hong-Ou-Mandel visibilities are defined as ${{\mathscr{V}}}_{ij,pq}=({{\mathscr{P}}}_{ij,pq}^{{\rm{d}}}-{{\mathscr{P}}}_{ij,pq}^{{\rm{q}}})/{{\mathscr{P}}}_{ij,pq}^{{\rm{d}}}$. Here ${{\mathscr{P}}}_{ij,pq}^{{\rm{d}}}$ is the probability for two distinguishable particles, obtained for a relative delay Δ*τ* much larger than the coherence time, and ${{\mathscr{P}}}_{ij,pq}^{{\rm{q}}}$ is the probability for two indistinguishable photons (Δ*τ* = 0). These quantities are related to the matrix elements of ${\mathscr{U}}$ as ${{\mathscr{P}}}_{i,j}={|{{\mathscr{U}}}_{j,i}|}^{2}$, ${{\mathscr{P}}}_{ij,pq}^{{\rm{d}}}={|{{\mathscr{U}}}_{p,i}{{\mathscr{U}}}_{q,j}|}^{2}+{|{{\mathscr{U}}}_{q,i}{{\mathscr{U}}}_{p,j}|}^{2}$ and ${{\mathscr{P}}}_{ij,pq}^{{\rm{q}}}={|{{\mathscr{U}}}_{p,i}{{\mathscr{U}}}_{q,j}+{{\mathscr{U}}}_{q,i}{{\mathscr{U}}}_{p,j}|}^{2}$. The training set is thus composed by the measured values ${\tilde{{\mathscr{P}}}}_{i,j}$ and ${\tilde{{\mathscr{V}}}}_{ij,pq}$, with associated gaussian 1*σ* experimental errors ${\rm{\Delta }}{\tilde{{\mathscr{P}}}}_{i,j}$ and ${\rm{\Delta }}{\tilde{{\mathscr{V}}}}_{ij,pq}$.

Let us now describe how to adapt the principles of genetic evolution to develop the actual algorithm (described in Supplementary Note 1). In this scenario, the group of individuals is a set of unitary transformations. At the initial step, the population Φ is composed of *N* randomly-chosen individuals *E*_*l*_: Φ = {*E*_1_, …, *E*_*N*_}. Every individual *E*_*l*_ has to be completely determined by a set of real parameters, that represent its DNA. It is thus necessary to identify a suitable choice to decompose a unitary transformation in a set of elementary blocks. At a first glance, one could consider the elements of the unitary transformation (moduli and phases) to compose the DNA of the individuals. However, this is not an appropriate choice since the generation of new offsprings from a random recombination of the parents according to this mechanism can lead to a non-unitary matrix. A better approach is obtained by exploiting the result by Reck *et al*.^49^, which showed that it is possible to decompose any linear transformation in a network composed of phase shifters (PS) and beam splitters (BS) (see Fig. 1a). A single gene ${G}_{k}^{l}=({t}_{k}^{l},{\alpha }_{k}^{l},{\beta }_{k}^{l})$ of the unitary’s DNA is composed of a PS-PS-BS set, defined by the transmittivity ${t}_{k}^{l}\in \mathrm{[0},1)$, and by the phases ${\alpha }_{k}^{l},{\beta }_{k}^{l}\in [0,\pi ]$. The DNA of an individual is thus represented by an ordered vector ${E}_{l}=\{{G}_{1}^{l},{G}_{2}^{l},\ldots ,{G}_{M}^{l}\}$, where $M={\sum }_{g=1}^{m-1}\,g=m(m-\mathrm{1)}/2$. The global unitary ${{\mathscr{U}}}_{{E}_{l}}$ of the individual *E*_*l*_ can be obtained by multiplying the set of unitary matrices ${{\mathscr{U}}}_{k}^{l}$ describing the action of the *k*-th gene ${G}_{k}^{l}$. With such a parametrization, unitariety of the overall transformation is naturally guaranteed. Note that this decomposition does not necessarily represent the actual internal structure of the system, which may be in general unknown. Indeed, it represents a mathematical tool to parametrize a unitary matrix as the combination of independent genes.

Once defined the decomposition of a unitary transformation in elementary units, it is necessary to implement the mechanism of genetic evolution. The first ingredient is the *fitness function* $f(E)\in {\mathbb{R}}:\mathrm{[0},\infty )$, which quantifies the survival probability of a given individual. In our case *f*(*E*) is chosen to be inversely proportional to the distance between the training set and theoretical predictions. Assuming ${{\mathscr{P}}}_{i,j}^{{E}_{l}}$ and ${{\mathscr{V}}}_{ij,pq}^{{E}_{l}}$ to be one-photon and two-photon predictions calculated from ${{\mathscr{U}}}_{{E}_{l}}$, we define the fitness function as *f*(*E*_*l*_) = 1/*χ* ^2^, where ${\chi }^{2}={\chi }_{{\mathscr{P}}}^{2}+{\chi }_{{\mathscr{V}}}^{2}$ is the chi-square function composed by two terms

1 $${\chi }_{{\mathscr{P}}}^{2}=\sum _{i,j}\,\frac{{({\tilde{{\mathscr{P}}}}_{i,j}-{{\mathscr{P}}}_{i,j}^{{E}_{l}})}^{2}}{{\rm{\Delta }}{\tilde{{\mathscr{P}}}}_{i,j}^{2}};{\chi }_{{\mathscr{V}}}^{2}=\sum _{i,j,p,q}\,\frac{{({\tilde{{\mathscr{V}}}}_{ij,pq}-{{\mathscr{V}}}_{ij,pq}^{{E}_{l}})}^{2}}{{\rm{\Delta }}{\tilde{{\mathscr{V}}}}_{ij,pq}^{2}}.$$

In other words, the fitness *f*(*E*_*l*_) represents the quality of the solution *E*_*l*_. At the beginning of each step of the evolution, the individuals are sorted in decreasing order according to their fitnesses. The second half of the population corresponding to the lowest fitness is removed, and replaced with a new set of individuals according to the *crossover function*. The latter governs the reproduction mechanism within the population. Two individuals *E*_*A*_ and *E*_*B*_ generate one child *E*_*C*_ whose DNA is composed of half genes from parent *E*_*A*_ and the other half from parent *E*_*B*_, chosen randomly. In the crossover mechanism, the position of the genes to be inherited from *E*_*A*_ or *E*_*B*_ occupy the same place in the child’s DNA sequence. Finally, the third ingredient is the *mutation process*. For any iteration of the algorithm any gene ${G}_{k}^{l}$ has a probability *γ* (called mutation rate) of being replaced by a new random triple $\{{\tilde{t}}_{k}^{l},{\tilde{\alpha }}_{k}^{l},{\tilde{\beta }}_{k}^{l}\}$. One of the main advantages of genetic algorithms^38–40^ with respect to other methods is their capability of performing an exhaustive search in the parameters space. This is achieved by controlling the mutation rate, and by including a meaningful sampling probability for the DNA elements to avoid exploring sparser regions of the parameters space. Furthermore, genetic algorithms are particularly suitable to be implemented using parallel computation strategies, thus enabling to exploit this approach to significantly reduce the computational time. The price to pay with respect to other methods is the reduction of the system governability, since the evolution is not deterministic.

Before applying the developed algorithm, it is necessary to determine in a training stage the optimal combination of hyperparameters, that cannot be derived a priori and may depend on the dimension *m* of the network. It is thus possible to evaluate the convergence of the algorithm by tuning the hyperparameters with numerically simulated data (see Supplementary Note 2 and Supplementary Fig. 1). Indeed, an inappropriate choice of the hyperparameters can prevent the algorithm to reach the desired global minimum. The two most important hyperparameters are mutation rate and population size. The former is a crucial parameter, since its incorrect setting may lead the algorithm to avoid convergence to the global minimum. Indeed, an exceedingly high mutation frequency would reduce the search process to a random walk in the space of solutions, while an extremely small value would prevent the algorithm to reach the global maximum of *f*(*E*). Population size has to be adjusted to avoid an unnecessary large population, which contains redundant elements and may significantly increase the number of iterations to reach convergence, or a too small population, not allowing to explore a sufficient set of gene combinations. Other less crucial hyperparameters involve: the number of iterations to wait until packing is performed, and the number *N*_1_ of unitaries to pick from the analytic algorithm (only if they are included in the initial population).

The convergence of the algorithm once determined the correct set of hyperparameters has been tested with numerically simulated data, showing that the algorithm is able to reach a value of the *χ* ^2^ close to its expectation value (equal to the number of degree of freedom *ν*, see Supplementary Note 2 and Supplementary Fig. 1). More specifically, we observe that a single set of hyperparameters can be employed for all unitary matrices at fixed *m*, while it is likely that by further increasing the interferometer dimension *m*, the set of hyperparameters has to be tuned to optimize and guarantee convergence of the algorithm.

### Experimental results

We tested the genetic algorithm by reconstructing the transformation induced by a 7-mode integrated interferometer, fabricated in a borosilicate glass substrate by means of the femtosecond laser waveguide writing^46,47^ technique. This approach exploits the permanent and localized increase in the refraction index obtained by nonlinear absorption of focused femtosecond pulses, thus directly writing waveguides in the material. The internal structure of the implemented interferometer is shown in Fig. 1d, and is composed by a network of symmetric 50–50 directional couplers and a phase pattern. We observe that the internal structure of the interferometer is different from the triangular structure adopted in the genetic algorithm, being the latter only a mathematical tool.

Single-photon and two-photon input states, necessary to measure the data set of the algorithm, were prepared by a spontaneous parametric down conversion source. The 750 mW pump beam at *λ*_*P*_ = 392.5 nm is obtained by second-harmonic generation of a *λ* = 785 nm pulsed Ti:Sa laser source, with 76 MHz repetition rate and Δ*τ* = 250 fs pulse duration. The photon source is a type-II, 2 mm length BBO crystal (Beta-Barium Borate), which generates pairs of photons with opposite polarization. Photons after generation are spectrally selected by 3 nm interference filters, analyzed in polarization, collected in single-mode fibers, and then propagated through two independent delay lines to adjust the time difference Δ*τ* between the input particles. Then, after fiber polarization compensation, the generated photons are injected in the input modes of the interferometer through a single-mode fiber array, and are then collected by a multi-mode fiber array before detection with a set of single-photon avalanche photodiodes (APD). Output single photon counts and two-fold coincidences are collected by an electronic acquisition system. The overall apparatus (source, interferometer and detectors) does not require phase stabilization. Indeed, due to the integrated implementation of the unitary transformation, the interferometer is intrinsically stable with respect to internal phases. Furthermore, injection and detection of Fock states renders the system insensitive to fluctuating phases at the input and at the output of the device, differently from other methods relying on classical light^35^.

**Figure 2:**
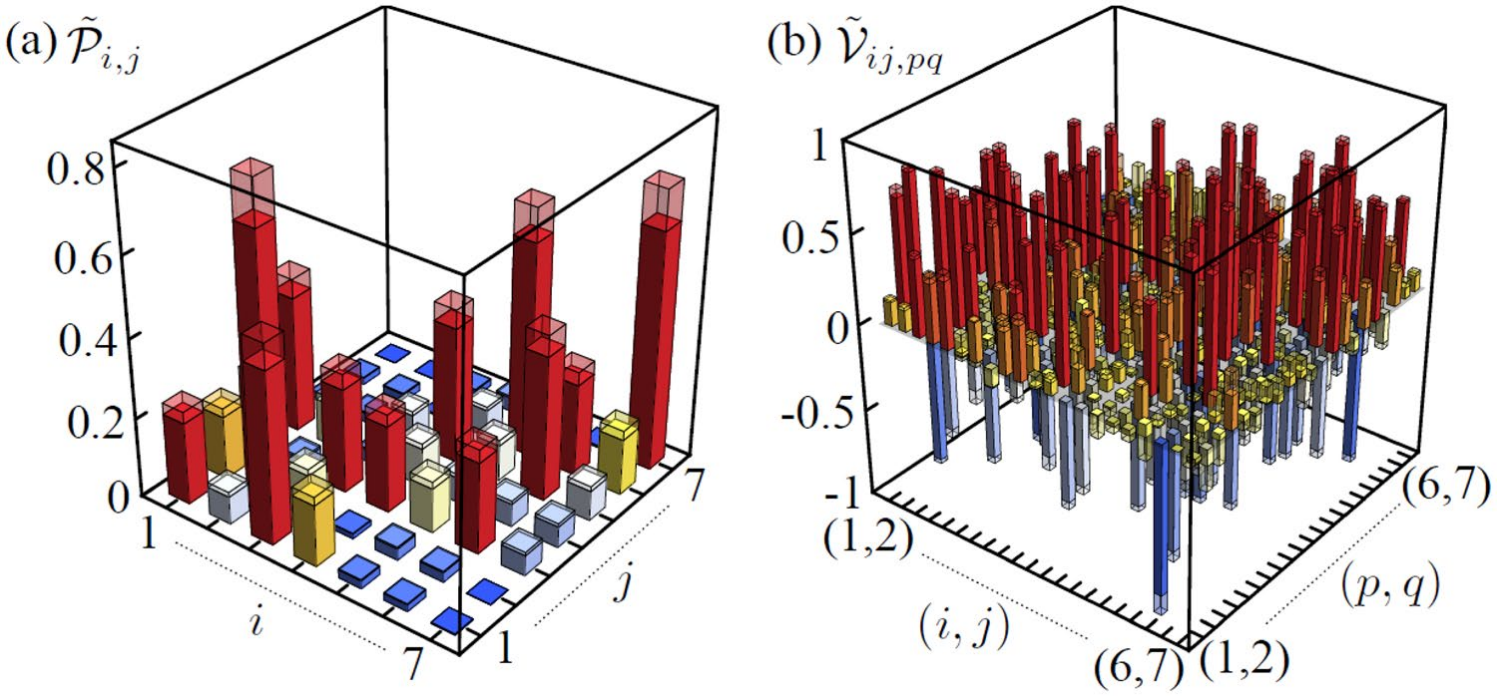
(**a**) Measured single-photon probabilities ${\tilde{{\mathscr{P}}}}_{i,j}$. (**b**) Measured two-photon Hong-Ou-Mandel visibilities ${\tilde{{\mathscr{V}}}}_{ij,pq}$. Shaded regions correspond to the experimental errors.

The learning method based on the genetic approach has been applied to the 7-mode chip. The complete set of experimental measurements consists of *d*_1_ = 49 = *m* ^2^ single-photon transition probabilities ${\tilde{{\mathscr{P}}}}_{i,j}$ and *d*_2_ = 441 = *m* ^2^(*m* − 1)^2^/4 two-photon Hong-Ou-Mandel visibilities ${\tilde{{\mathscr{V}}}}_{ij,pq}$, corresponding to an overall amount of *d* = *d*_1_ + *d*_2_ = 490 data. The complete set of collected experimental data is reported in Fig. 2. The two-photon visibilities ${\tilde{{\mathscr{V}}}}_{ij,pq}$, insensitive to photon losses, are estimated by recording the input-output two-fold coincidences ${\tilde{{\mathscr{P}}}}_{ij,pq}({\rm{\Delta }}\tau )$ as a function of the relative delay Δ*τ* between the input photons. The resulting pattern is analyzed by performing a best fit according to the function ${\tilde{{\mathscr{P}}}}_{ij,pq}({\rm{\Delta }}\tau )=C(1+{\tilde{{\mathscr{V}}}}_{ij,pq}{e}^{-{\sigma }^{2}{(\tau -{\tau }_{0})}^{2}})$, where $(C,{\tilde{{\mathscr{V}}}}_{ij,pq},\sigma ,{\tau }_{0})$ are free-parameters and *τ* is the independent variable. Experimental errors on the single-photon probabilities ${\rm{\Delta }}{\tilde{{\mathscr{P}}}}_{i,j}$ and on the two-photon coincidences ${\tilde{{\mathscr{P}}}}_{ij,pq}({\rm{\Delta }}\tau )$ are due to the Poissonian statistics of the measured events, while errors on the visibilities ${\tilde{{\mathscr{V}}}}_{ij,pq}$ are obtained from the fitting procedure.

**Figure 3:**
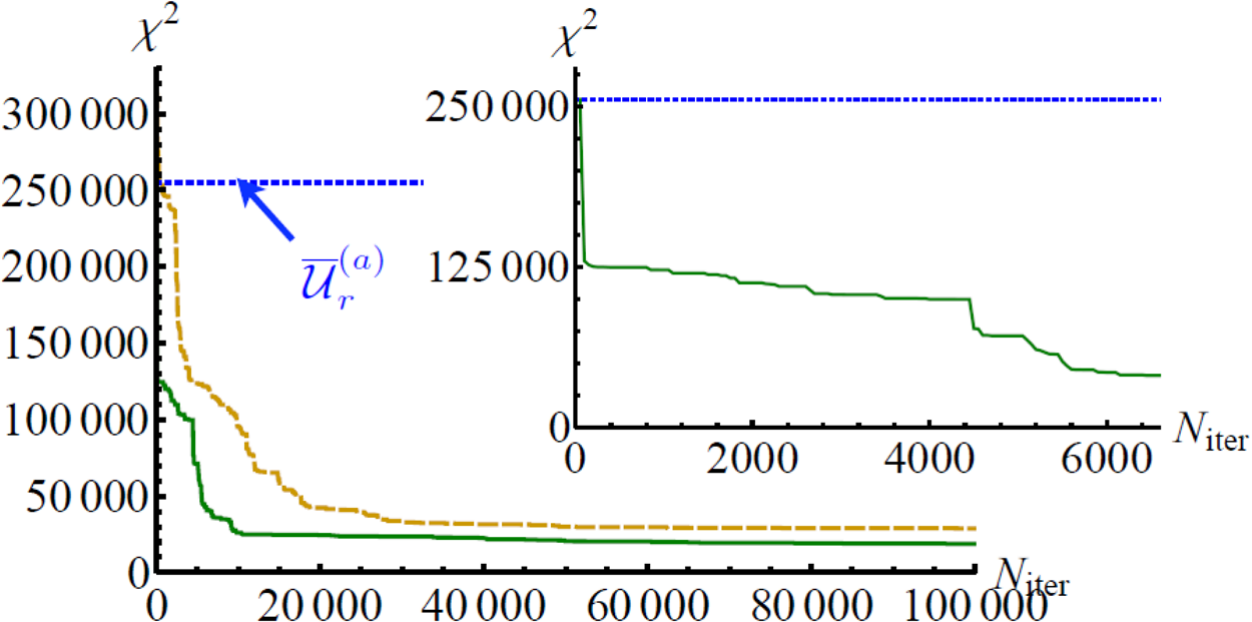
Evolution of the minimum *χ* ^2^ in the genetic pool through the running time of the algorithm, as a function of the number of iterations. Dark yellow dashed lines: the starting point is provided by a random set of individuals. Green solid lines: the initial population includes the best *N*_1_ = 20 unitaries obtained with the analytic method. Horizontal blue dotted lines: best *χ* ^2^ obtained with the analytic method. Inset: highlight for *N*_iter_ ∈ [0; 6600] of the green curve. The *χ* ^2^ values achieved during the algorithm iterations correspond to a range $40\mathop{ < }\limits_{ \tilde {}}{\chi }_{\nu }^{2}\mathop{ < }\limits_{ \tilde {}}585$ for the reduced ${\chi }_{\nu }^{2}$.

The genetic algorithm maximizes the fitness function *f*(*E*_*l*_) [Eq. (1)] between the training set and the predictions ${{\mathscr{P}}}_{i,j}^{{E}_{l}}$ and ${{\mathscr{V}}}_{ij,pq}^{{E}_{l}}$ obtained from the unitary ${{\mathscr{U}}}_{{E}_{l}}$ belonging to the population of the genetic algorithm. The starting point of the protocol is a random population of *N* = 100 unitaries. In Fig. 3 we report the evolution of the best *χ* ^2^ in the genetic pool during the running time of the algorithm. We observe that an almost stable value of the *χ* ^2^ is obtained after ${N}_{{\rm{iter}}}\sim 60000$ iterations. The genetic approach can be improved by modifying the starting point. At the initial step, a subset of *N*_1_ unitaries can be chosen starting from the algorithm of ref.^34^. With that method, a minimal set of two-photon data is exploited to retrieve analytically the elements of the unitary matrix. This approach can be extended by considering that *m* ^2^ independent estimates of ${\mathscr{U}}$ can be obtained by recording the full set of single- and two-photon measurements, and by appropriately permuting the mode indexes^17^ to select *m* ^2^ independent minimal data sets. For the genetic algorithm, we then choose the *N*_1_ = 20 unitaries (among the *m* ^2^ = 49 possible matrices) presenting the lower values of *χ* ^2^ (higher fitnesses). This provides a reasonable starting point for the genetic pool. Finally, the remaining subset of *N*_2_ = 80 are randomly generated from the Haar measure. In such a way, the algorithm reaches convergence after a smaller number of iterations ${N}_{{\rm{iter}}}\sim 40000$, corresponding to a computational time of *t*~1 h on a laptop (see Fig. 3). The convergence of the genetic algorithm is confirmed by the decrease of *χ* ^2^ from the starting value ${{\rm{\min }}}_{{{\mathscr{U}}}_{r}^{(a)}}\,{\chi }^{2}\sim 255000$, obtained from the best unitary ${\overline{{\mathscr{U}}}}_{r}^{(a)}$ of the analytic approach, to a final value of ${\chi }_{r,(g)}^{2}\sim 17096$, leading to an improvement of one order of magnitude. Given the number of degrees of freedoms *ν* for this problem size $$\nu ={m}^{2}+{(\begin{array}{c}m\\ n\end{array})}^{2}-3m(m-\mathrm{1)/2}=427$$, the final result corresponds to a value of the reduced ${\chi }_{\nu }^{2}={\chi }^{2}/\nu \sim 40$. This value of the reduced ${\chi }_{\nu }^{2}$ indicates that the adopted model for the unitary transformation and for the input photons needs to be improved. Indeed, the algorithm employs as internal structure the general decomposition given by Reck’s lemma^49^, and does not take into account the actual layout of the device. In particular, internal and output losses can lead to non-unitary behavior and are not taken into account by the present model. Furthermore, the input photons for two-photon measurements are not perfectly indistiguishable. The value of the reduced ${\chi }_{\nu }^{2}$ can be thus improved by including these features in the fitness evaluation. For instance, partial photon indistinguishability can be included as an additional parameter *p* in the *χ* ^2^ that reduces the value of the two-photon visibilities. By adding it only in the final calculation of the *χ* ^2^, the latter diminishes from ${\chi }^{2}\sim 17096$ to ${\chi }^{2}\sim 14067$, corresponding to a reduced value ${\chi }_{\nu }^{2}\sim 33$ (for a value of *p* = 0.95 pre-characterized with an Hong-Ou-Mandel interference measurement). Improved results are expected if this parameter is included directly in the fitness evaluation.

As an additional figure of merit, we consider the similarities ${S}_{r}^{(a)}$ between the experimental two-photon visibilities and the predictions obtained from the analytic unitaries ${{\mathscr{U}}}_{r}^{(a)}$, according to the definition ${S}_{r}^{(a)}=1-{\sum }_{i,j,p,q}\,|{\tilde{{\mathscr{V}}}}_{ij,pq}-{{\mathscr{V}}}_{ij,pq}^{r,(a)}|/\mathrm{(2}{d}_{2})$ (and analogous definition for the genetic approach). The similarity ${S}_{r}^{(g)}$ obtained for the output unitary ${{\mathscr{U}}}_{r}^{(g)}$ from the genetic algorithm, equal to ${S}_{r}^{(g)}=0.957\pm 0.001$, clearly outperforms the maximum value obtained from the analytic algorithm: ${{\rm{\max }}}_{{{\mathscr{U}}}_{r}^{(a)}}\,{S}_{r}^{(a)}=0.920\pm 0.001$. This suggests that, while a direct analitic inversion^34^ from ${{\mathscr{V}}}_{ij,pq}$ to ${\mathscr{U}}$ permits to obtain *m* ^2^ independent estimates each requiring a smaller amount of data (≈2 *m* ^2^), the adoption of a larger training set and the capability of taking into account experimental errors (in the *χ* ^2^) in the genetic approach permits to increase the robustness with respect to experimental noise.

Finally, we observe that the decrease of *χ* ^2^ occurs with two different trends (see inset of Fig. 3). Smooth variations are due to the crossover mechanism between members of the population, converging to the best possible unitary within the available genetic pool. Conversely, fast jumps in *χ* ^2^ are due to random mutations in the genetic pool.

**Figure 4:**
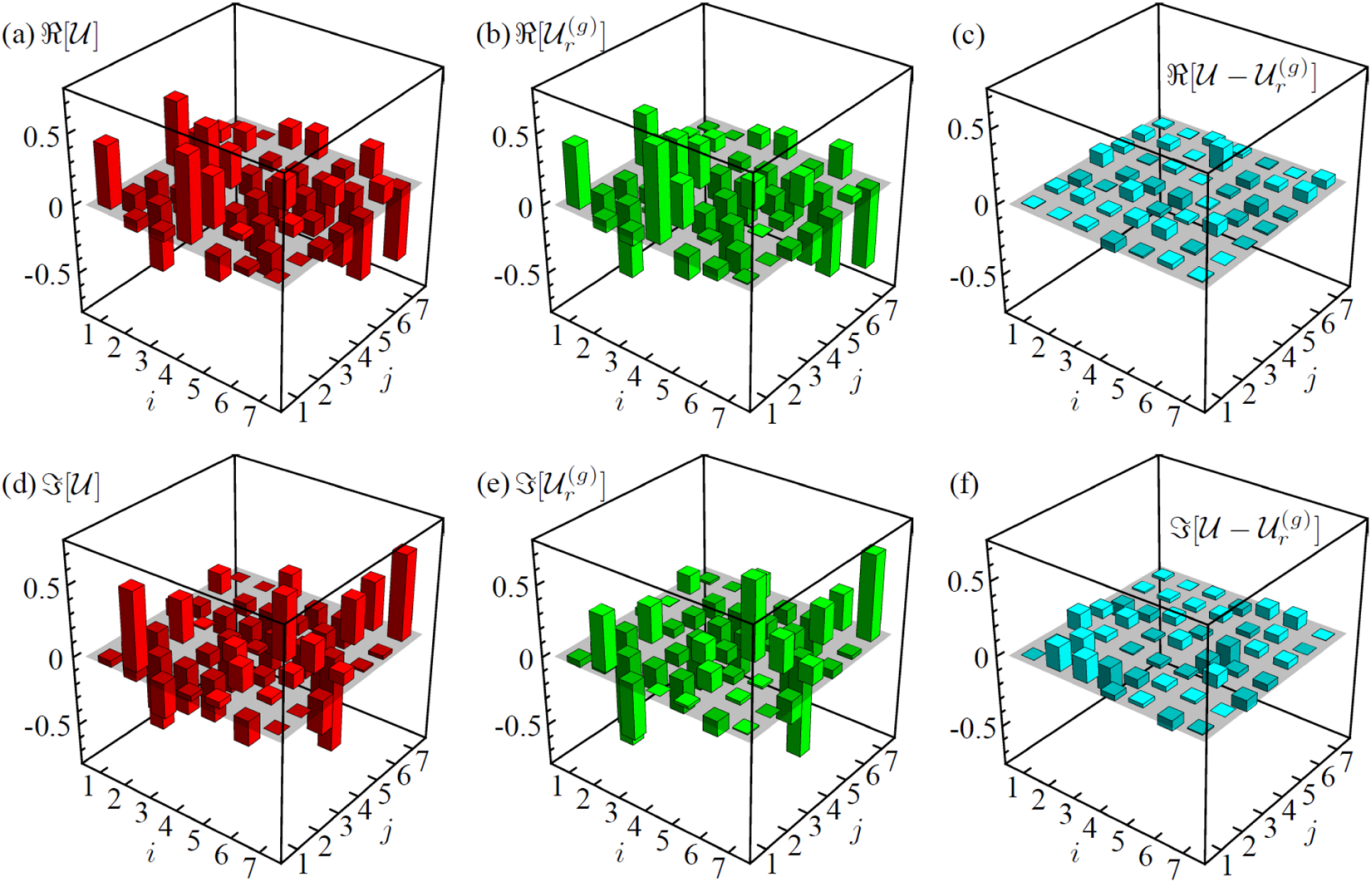
(**a**) Real and (**d**) imaginary parts of the theoretical unitary matrix ${\mathscr{U}}$. (**b**) Real and (**e**) imaginary parts of the reconstructed matrix ${{\mathscr{U}}}_{r}^{(g)}$. (**c**) Real and (**f**) imaginary parts of the difference ${\mathscr{U}}-{{\mathscr{U}}}_{r}^{(g)}$.

The results for the obtained transformation are reported in Fig. 4 and in Supplementary Note 3, where the output unitary of the genetic algorithm ${{\mathscr{U}}}_{r}^{(g)}$ is compared with the theoretical one ${\mathscr{U}}$. The latter is calculated from fabrication parameters according to the layout of Fig. 1d. This comparison indicates how close the implemented interferometer is with respect to the ideal one. A quantitative parameter is the gate fidelity between ${\mathscr{U}}$ and ${{\mathscr{U}}}_{r}^{(g)}$, defined as ${F}_{r}^{(g)}=|{\rm{Tr}}[{{\mathscr{U}}}^{\dagger }{{\mathscr{U}}}_{r}^{(g)}]|/m$. The value obtained for the implemented interferometer is ${F}_{r}^{(g)}=0.975\pm 0.013$, thus showing the quality of the fabrication process. The error on the gate fidelity ${F}_{r}^{(g)}$ has been estimated by a *M* = 10000 Monte-Carlo simulation of unitary reconstructions with the analytic method.

Further optimizations of the protocol can be envisaged. The *χ* ^2^ function in the fitness may be replaced with a weighted function ${\chi }_{w}^{2}=w{\chi }_{{\mathscr{P}}}^{2}+\mathrm{(1}-w){\chi }_{{\mathscr{V}}}^{2}$. We then performed the reconstruction method for different values of the weight *w*, observing that for the present data the best choice is obtained for the symmetric case *w* = 0.5. The optimal weight *w* can nevertheless vary with the dimension *m* of the network. Additionally, the number of unitaries *N*_1_ taken at the initial step from the analytic algorithm can be optimized depending on the problem size. Room for optimization can be also found by checking the possibility of adding childs with random permutations in the location of the genes. Finally, as previously discussed the reported method can be modified to exploit knowledge on the internal structure of the device, including internal losses due to propagation and to the directional couplers. In the experimental case shown above, the universal structure can be replaced by the actual structure of the interferometer, thus redefining its DNA.

## Conclusions

In this article we have described an approach to learn an unknown linear optical process ${\mathscr{U}}$ by exploiting a specifically tailored genetic algorithm. We have then tested this approach for the reconstruction of an unknown 7 × 7 integrated linear optical interferometer built by the femtosecond laser-writing technique. The experimental results show that this methodology is suitable for the characterization of linear optical networks. The involved resources (number of parameters for the DNA and size of the data set) scale polynomially with the size *m* of the network. Furthermore, the evaluation of the fitness functions requires resources scaling polynomially as *m* ^4^. Thus, this approach could be suitable to be employed on systems with increasing number of modes, with applications in different contexts such as quantum simulation and quantum interferometry. Several perspectives can be envisaged by applying these genetic approaches in the context of learning unknown patterns^50^ or general Hamiltonian evolutions^23^. The algorithmic approach itself may be adapted to progressively change the parameters of its evolution or the measured data set sequence depending on the results of the previous steps.

## Electronic supplementary material

Supplementary Information
