# Supplementary material for: Learning an unknown transformation via a genetic approach
Source: Sci Rep. 2017 Oct 30;7:14316. doi: 10.1038/s41598-017-14680-7 (PMC5662785; doi:10.1038/s41598-017-14680-7)
Supplement: Supplementary file 1 — Supplementary Information [file 41598_2017_14680_MOESM1_ESM.pdf]

Supplementary Information - Learning an unknown transformation via a genetic approach

Nicolò Spagnolo,[1,*] Enrico Maiorino,[1] Chiara Vitelli,[1] Marco Bentivegna,[1] Andrea Crespi,[2,3]
Roberta Ramponi,[2,3] Paolo Mataloni,[1] Roberto Osellame,[2,3] and Fabio Sciarrino[1,†]

[1]*Dipartimento di Fisica, Sapienza Università di Roma, Piazzale Aldo Moro 5, I-00185 Roma, Italy*
[2]*Istituto di Fotonica e Nanotecnologie, Consiglio Nazionale delle Ricerche (IFN-CNR), Piazza Leonardo da Vinci, 32, I-20133 Milano, Italy*
[3]*Dipartimento di Fisica, Politecnico di Milano, Piazza Leonardo da Vinci, 32, I-20133 Milano, Italy*

SUPPLEMENTARY NOTE 1: THE ALGORITHM

The $m \times m$ interferometer is injected with single photon and two-photon input states to obtain the starting data sets. From single photon measurements the input/output interferometer couplings $\tilde{\mathcal{P}}_{i,j}$ are obtained (with corresponding errors $\Delta\tilde{\mathcal{P}}_{i,j}$), while two-photon measurements give rise to $h = \binom{m}{2}^2$ possible Hong-Ou-Mandel interference patterns, quantified by the corresponding dip (or peak) visibilities $\tilde{\mathcal{V}}_{ij,pq}$ with errors $\Delta\tilde{\mathcal{V}}_{ij,pq}$.

The genetic algorithm, which aims at learning the unitary transformation $\mathcal{U}_r$ starting from the collected data set, is structured as follows.

1. A distribution of N DNA sequences, representing N different $m \times m$ unitary matrices, is generated. The parameters $\{t_k^l, \alpha_k^l.\beta_k^l\}$ are drawn from appropriate distributions, so that the generated unitaries are distributed according to the Haar measure [S1]. An approximate form of these distributions have been evaluated numerically by sampling unitary matrices from the Haar measure. More specifically, the phase differences $\alpha_k^l - \beta_k^l$ are drawn from the uniform distribution, while the transmittivities t_k^l are drawn from a triangular one $u(t_i) = 2t_i$. The exact form of these distribution can be evaluated as shown in [S2]. The obtained set of N DNAs constitutes the population $\tilde{\Phi}_0 = \{\tilde{E}_1, ..., \tilde{E}_N\}$.

1′. The analytic method proposed in Ref. [S3] is applied to the experimental data. A set of m^2 independent estimates of the unitary [S4] is obtained, starting from this approach, by selecting appropriate subsets of the data and by performing permutations of the mode indexes. DNA sequences for the $N_1 = 20$ unitaries presenting higher fitnesses are then evaluted. Finally, N_1 elements of the population $\tilde{\Phi}_0$ obtained at step 1 are replaced by the N_1 candidates determined from the analytic method. The new set of N DNAs constitutes the initial population $\Phi_0 = \{E_1, ..., E_N\}$.

2. The population is sorted by decreasing fitness values, evaluated between the experimental data $(\tilde{\mathcal{P}}_{i,j}, \tilde{\mathcal{V}}_{ij,pq})$ and the predictions $(\mathcal{P}_{i,j}^{E_l}, \mathcal{V}_{ij,pq}^{E_l})$ obtained from the matrices of the population, with $l = 1, \dots, N$. The new ordered population set is $\Phi_1 = \{E_1', ..., E_N'\}$.

3. The single-photon probabilities $\mathcal{P}^{E_1'}$ and the two-photon visibilities $\mathcal{V}^{E_1'}$ are calculated from the element E_1'. If $f(E_1') \geq \delta$ the algorithm halts and returns the solution matrix $U_{E_1'}$. More specifically, the unitary matrix $U_{E_1'}$ is obtained from the conversion function $T(E_1')$ which relates the genetic code to the corresponding unitary transformation [S5].

4. The second half of the population, consisting of the individuals with lowest fitness values, is removed. The resized population Φ_2 is the set $\Phi_2 = \{E_1', ..., E_{N/2}'\}$.

5. Crossover is applied between two randomly chosen individuals. The corresponding generated offspring is added to the population set Φ_2. This operation is iterated with other couples of individuals until the number of elements of Φ_2 is N. The result of this mechanism is a new population $\Phi_3 = \{E_1', ..., E_{N/2}', \bar{E}_{N/2+1}, ...\bar{E}_N\}$, where the elements $\bar{E}_l$ are the newly-generated individuals.

6. During the evolution of the system, several individuals with the identical DNA (clones) corresponding to the element with highest fitness may spread in the population. This effect causes a steady depletion of the gene pool, which in turn leads to an early convergence of the algorithm to a local maximum of $f(E)$. To avoid this effect two countermeasures have been adopted: (i) ***Random Offspring Generation*** [S6], which imposes that crossover betweeen two clones generates a child with random DNA, and (ii) ***Packing***, which consists in identifying clusters of clones in the population every q iteration. For each of these clusters, all the elements except one are removed and the population is filled by randomly generated new individuals.

---

* nicolo.spagnolo@uniroma1.it
† fabio.sciarrino@uniroma1.it

7. For each element $l = 2, ..., N$, mutation is applied with probability γ. The index l starts from the value 2 to avoid a mutation on the individual with highest fitness in the population. This constraint is commonly refereed to as *Elitism*. A new population $\Phi_4 = \{E''_1, ..., E''_N\}$ is obtained.

8. Steps 2-7 are iterated starting from the new population Φ_4 until the halting condition is reached at step 3.

SUPPLEMENTARY NOTE 2: ALGORITHM CONVERGENCE

To characterize the performance of the developed genetic algorithm, we have performed numerical simulations for different circuit size with simulated data. More specifically, for each tested size we generated $N_{\text{unit}} = 50$ different Haar-random unitary matrices. For each matrix, the complete set of single-photon probabilities and two-photon visibilities is calculated. To include the effect of statistical errors corresponding to a finite size experimental data sample for each quantity, noisy data are simulated by generating random numbers following a Gaussian distribution with μ equal to the exact value, and σ equal to the simulated noise. We employed a value of the (relative) noise equal to 3% for single-photon probabilities, and 5% for the two-photon visibilities.

The simulated noisy data are fed into the genetic algorithm to learn the unitary transformation. The obtained results for $m = 4$, $m = 5$ and $m = 6$ interferometers are shown in Supplementary Fig. 1 and compared to what is obtained with the analytic approach (which is employed as seed for the genetic approach) and with a numerical derivative-based minimization routine (adopting the solution of the analytic method as a starting point). More specifically, we report the histograms of the reduced χ^2_ν (that is, the χ^2 divided by the number of degrees of freedom ν). We observe that the conventional numerical routine and the genetic approach provide comparable performances in terms of achieved χ^2_ν, with value close to 1. On the other side, the analytic approach in general fails to capture the optimal solution in the presence of statistical noise.

Note that the same set of parameters (mutation rate, population size, ...) is employed for all unitary matrices at a given size m. Furthermore, in these simulations a single set of parameters is shown to be effective for all investigated m. However, it is likely that by further increasing the interferometer dimension m, the set of hyperparameters has to be tuned to optimize and guarantee convergence of the algorithm.

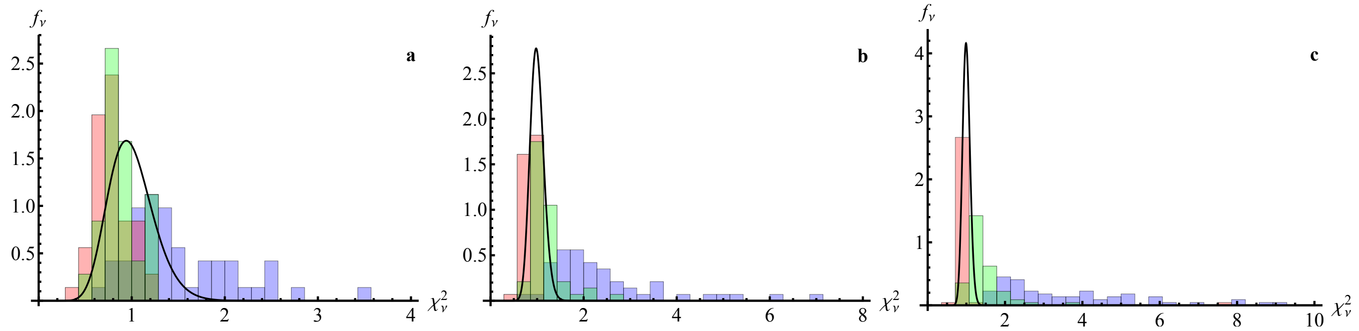

Supplementary Figure 1. **Numerical simulations for algorithm convergence.** Histogram of reduced χ^2_ν obtained at the end of the reconstruction algorithm with different approaches. For each size, the histogram refers to $N_{\text{unit}} = 50$ different unitary matrices with simulated noise. Blue: best solutions obtained with the analytic approach. Green: solutions obtained through genetic algorithm with analytic unitaries as seed. Red: solutions obtained through numerical minimization routine with the best analytic unitary as starting point. Black line: theoretical distribution for the reduced χ^2_ν for each problem size, provided for comparison. **a** $m = 4$. **b** $m = 5$. **c** $m = 6$.

SUPPLEMENTARY NOTE 3: EXPECTED AND RECONSTRUCTED UNITARY MATRICES

Here we report the unitary matrix corresponding to the interferometer design $\mathcal{U}$ and the one obtained from the reconstruction with the genetic approach $\mathcal{U}_r^{(g)}$, shown in Fig. 4 of the main text. The expected unitary matrix is calculated by exploiting from the actual internal structure of device, shown in Fig. 1 d, which is composed by a network of symmetric 50/50 beam-splitters interspersed by static relative phases between the modes. The fabrication phases for each layer are reported in Supplementary Table 1.

	L_1	L_2	L_3	L_4
W_1	1.5253	0.6993	2.7776	1.8087
W_2	2.6182	1.5267	2.9560	1.6449
W_3	1.9217	2.8131	0.6705	1.7497
W_4	0.7217	0.4718	0.9392	1.5706
W_5	2.9256	1.3138	2.1079	2.8032
W_6	0.4974	1.4759	0.3152	0.7684
W_7	2.0089	2.9217	1.3470	2.0250

Supplementary Table 1. Fabrication phases of the implemented device. On each column we report the phases of a given layer L_i for the waveguides W_j.

The expected unitary $\mathcal{U}$ has real part:

$$\Re[\mathcal{U}] = \begin{pmatrix} 0.440067 & -0.144084 & -0.213019 & 0.478544 & 0.148792 & 0.0954517 & 0. \\ -0.0824973 & -0.483843 & -0.113649 & 0.0270831 & -0.0954517 & 0.148792 & 0. \\ -0.0668478 & 0.0540365 & 0.520786 & 0.257786 & -0.168401 & -0.0722028 & 0.159266 \\ 0.59962 & 0.362828 & -0.192912 & -0.408267 & -0.295213 & -0.137872 & 0.192703 \\ -0.171234 & 0.0439199 & -0.252021 & 0.211038 & -0.173805 & -0.344251 & -0.0907635 \\ -0.0439199 & -0.171234 & 0.0840009 & -0.104909 & 0.1454 & -0.320468 & 0.217302 \\ 0. & 0. & -0.0825909 & -0.235963 & -0.45092 & 0.131444 & -0.533947 \end{pmatrix} \quad (1)$$

and imaginary part:

$$\Im[\mathcal{U}] = \begin{pmatrix} -0.0460727 & -0.257413 & -0.605506 & 0.116951 & -0.0954517 & 0.148792 & 0. \\ 0.555327 & -0.531634 & 0.302887 & 0.0133133 & -0.148792 & -0.0954517 & 0. \\ -0.247407 & -0.332066 & -0.0992346 & -0.391134 & -0.136487 & -0.453793 & 0.192703 \\ -0.0360857 & -0.298176 & 0.126105 & -0.0298965 & -0.14752 & -0.0865235 & -0.159266 \\ 0.0439199 & 0.171234 & -0.0760492 & 0.458373 & -0.22598 & -0.600826 & -0.211058 \\ -0.171234 & 0.0439199 & 0.101443 & 0.203086 & -0.689667 & 0.31566 & 0.348654 \\ 0. & 0. & -0.235963 & 0.0825909 & -0.0146029 & -0.0272751 & 0.606908 \end{pmatrix} \quad (2)$$

The reconstructed unitary matrix $\mathcal{U}_r^{(g)}$ with the genetic approach has real part:

$$\Re[\mathcal{U}_r^{(g)}] = \begin{pmatrix} 0.4355 & -0.1775 & -0.1611 & 0.3850 & 0.1002 & 0.04614 & -0.01467 \\ -0.08493 & -0.5299 & -0.1084 & 0.07069 & -0.08722 & 0.09845 & -0.004452 \\ -0.09533 & 0.02920 & 0.4440 & 0.3112 & -0.2021 & -0.07885 & 0.1113 \\ 0.6582 & 0.3075 & -0.1640 & -0.3307 & -0.2972 & -0.2983 & 0.1659 \\ -0.1592 & -0.02603 & -0.2743 & 0.1996 & -0.1505 & -0.2898 & -0.08246 \\ -0.07511 & -0.1536 & 0.005676 & -0.06572 & 0.2152 & -0.3605 & 0.2140 \\ -0.008678 & -0.00004971 & -0.07865 & -0.2236 & -0.4224 & 0.05521 & -0.5920 \end{pmatrix} \quad (3)$$

and imaginary part:

$$\Im[\mathcal{U}_r^{(g)}] = \begin{pmatrix} -0.04560 & -0.1994 & -0.7269 & 0.05497 & 0.002889 & 0.1366 & -0.01895 \\ 0.3917 & -0.6540 & 0.2609 & -0.0613 & -0.09862 & -0.1233 & -0.001719 \\ -0.4036 & -0.1589 & -0.08890 & -0.3718 & -0.1918 & -0.4876 & 0.1664 \\ 0.0001499 & -0.2371 & 0.1527 & -0.09482 & -0.1162 & -0.1312 & -0.1089 \\ 0.01891 & 0.1362 & -0.05465 & 0.5653 & -0.0433 & -0.5648 & -0.2884 \\ -0.1041 & 0.02457 & 0.01568 & 0.2373 & -0.7350 & 0.2596 & 0.2622 \\ 0.008239 & -0.000001997 & -0.1609 & 0.1284 & -0.06000 & -0.02719 & 0.6042 \end{pmatrix} \quad (4)$$

SUPPLEMENTARY REFERENCES

[S1] Spengler, C., Huber, M. & Hiesmayr, B. C. A composite parameterization of unitary groups, density matrices and subspaces. *Journal of Mathematical Physics* **53**, 013501 (2012).
[S2] Russell, N. J., Chakhmakhchyan, L., OBrien, J. L. & Laing, A. Direct dialling of Haar random unitary matrices. *New J. Phys.* **19**, 033007 (2017).
[S3] Laing, A. & OBrien, J. L. arXiv:1208.2868v1 (2012).
[S4] Crespi, A. *et al.* Integrated multimode interferometers with arbitrary design for photonic boson sampling. *Nature Photonics* **7**, 545-549 (2013).
[S5] Reck, M., Zeilinger, A., Bernstein, H. J. & Bertani, P. Experimental realization of any discrete unitary operator. *Phys. Rev. Lett.* **73**, 58-61 (1994).
[S6] Rocha, M. & Neves, J. in Multiple Approaches to Intelligent Systems, edited by Springer (1999) pp. 127136.
